# Mobile Phone Surveys for Collecting Population-Level Estimates in Low- and Middle-Income Countries: A Literature Review

**DOI:** 10.2196/jmir.7428

**Published:** 2017-05-05

**Authors:** Dustin G Gibson, Amanda Pereira, Brooke A Farrenkopf, Alain B Labrique, George W Pariyo, Adnan A Hyder

**Affiliations:** ^1^ Department of International Health Johns Hopkins Bloomberg School of Public Health Baltimore, MD United States; ^2^ Berman Institute of Bioethics Johns Hopkins University Baltimore, MD United States

**Keywords:** survey methodology, cellular phone, interactive voice response, short messages service, computer-assisted telephone interview, mobile phone surveys

## Abstract

**Background:**

National and subnational level surveys are important for monitoring disease burden, prioritizing resource allocation, and evaluating public health policies. As mobile phone access and ownership become more common globally, mobile phone surveys (MPSs) offer an opportunity to supplement traditional public health household surveys.

**Objective:**

The objective of this study was to systematically review the current landscape of MPSs to collect population-level estimates in low- and middle-income countries (LMICs).

**Methods:**

Primary and gray literature from 7 online databases were systematically searched for studies that deployed MPSs to collect population-level estimates. Titles and abstracts were screened on primary inclusion and exclusion criteria by two research assistants. Articles that met primary screening requirements were read in full and screened for secondary eligibility criteria. Articles included in review were grouped into the following three categories by their survey modality: (1) interactive voice response (IVR), (2) short message service (SMS), and (3) human operator or computer-assisted telephone interviews (CATI). Data were abstracted by two research assistants. The conduct and reporting of the review conformed to the Preferred Reporting Items for Systematic Reviews and Meta-Analyses (PRISMA) statement.

**Results:**

A total of 6625 articles were identified through the literature review. Overall, 11 articles were identified that contained 19 MPS (CATI, IVR, or SMS) surveys to collect population-level estimates across a range of topics. MPSs were used in Latin America (n=8), the Middle East (n=1), South Asia (n=2), and sub-Saharan Africa (n=8). Nine articles presented results for 10 CATI surveys (10/19, 53%). Two articles discussed the findings of 6 IVR surveys (6/19, 32%). Three SMS surveys were identified from 2 articles (3/19, 16%). Approximately 63% (12/19) of MPS were delivered to mobile phone numbers collected from previously administered household surveys. The majority of MPS (11/19, 58%) were panel surveys where a cohort of participants, who often were provided a mobile phone upon a face-to-face enrollment, were surveyed multiple times.

**Conclusions:**

Very few reports of population-level MPS were identified. Of the MPS that were identified, the majority of surveys were conducted using CATI. Due to the limited number of identified IVR and SMS surveys, the relative advantages and disadvantages among the three survey modalities cannot be adequately assessed. The majority of MPS were sent to mobile phone numbers that were collected from a previously administered household survey. There is limited evidence on whether a random digit dialing (RDD) approach or a simple random sample of mobile network provided list of numbers can produce a population representative survey.

## Introduction

National and subnational surveys are important for monitoring disease burden, prioritizing resource allocation, and evaluating public health policies [1]. In low- and middle-income countries (LMICs), such surveys typically rely on face-to-face interviews conducted at the respondent’s household. Household surveys are conducted infrequently, typically due to high costs in personnel and transportation associated with household survey implementation and the face-to-face nature of data collection [2-5]. In addition, household surveys require considerable amounts of time for data collection, data management, and data analysis which impedes the speed at which data become publically available. A more frequent surveillance of population health would allow for a more timely evaluation of implemented public health policies and response to public health emergencies.

To address the high costs and time requirements associated with household surveys, higher income countries have developed and employed telephone surveys to collect population-level estimates of health and demographics [6-8]. As mobile phone ownership and access become more common globally, with 94 subscriptions per 100 inhabitants in developing countries [9], opportunities exist to leverage mobile-health technologies and communication channels to revolutionize the current methods of data collection in LMIC. Rather than conducting household surveys, respondents can now be interviewed over their own personal mobile phone through the use of short message service (SMS), interactive voice response (IVR), and computer-assisted telephone interviews (CATI) survey modalities; collectively called mobile phone surveys (MPS).

SMS surveys utilize text messages to send survey questions to participants’ mobile phones. Data are then collected from participants via SMS responses to these questions. Inherent in this survey modality is the requirement of a literate population, which may be challenging in some LMICs. IVR surveys counter the challenges in SMS surveys by using automated, prerecorded questions. With IVR surveys, respondents interact with a preprogrammed database which contains both questions and a series of preset answers which are linked to a specific numeric key, or numeric response on a touch-tone phone keypad (eg, “Press 1 for Yes”). CATI surveys most closely mimic a household survey by employing human interviewers or call centers. Interviewers follow a script provided by a software program to survey participants.

The purpose of this review was to document the current landscape of MPS being used for population-level data collection in LMICs, with a focus on IVR-, SMS-, and CATI-collected data and to identify key survey metrics, such as response and completion rates for each of the MPS modalities. Such a review is currently not available in the literature, and this comprises an important assessment of current knowledge for future research [10]. 

## Methods

We conducted a systematic search of the literature according to the Preferred Reporting Items for Systematic Reviews and Meta-Analyses (PRISMA) [11] in March-April of 2015 to find articles that used MPS for population-level data collection in LMICs. There were no restrictions on year that records were published. Using a predefined search strategy that included a range of terms for mobile phone, interactive voice response, text message, survey, questionnaire, and data collection, we searched the primary and gray literature using PubMed, Embase, Scopus, Global Health, Web of Science, Cochrane Reviews, and Proquest Digital Dissertations databases for article titles, key words, and abstracts pertaining to MPS. Search terms were uniquely created for each database to capitalize on the database’s classification of articles (see Multimedia Appendix 1).

Records that matched the search criteria or were preidentified as relevant articles before the literature review (n=6) were imported into RefWorks. The 6 preidentified articles were obtained through our knowledge of the World Bank’s initiative to promote MPS in LMICs. After applying an automatic filter for duplicates, the remaining abstracts and titles were manually filtered for additional study duplication and clearly irrelevant topics, such as nonhuman studies. Two research assistants conducted a primary screening of each record’s titles and abstracts. Primary inclusion criteria included the following: (1) SMS used to collect data from a respondent, (2) IVR used to collect data from a respondent, (3) CATI or call centers used to collect data from a respondent, (4) a combination of the above, (5) surveys where a respondent provided answers using their mobile phone. Primary exclusion criteria included the following: (1) mobile phone used by a human enumerator to conduct an in-person survey, (2) use of mobile phone for other activities but not data collection, or (3) no indication that mobile phones were used for data collection. Records were included for secondary screening if there was any uncertainty as to whether the article included an MPS.

Articles that met primary screening criteria underwent a secondary full-text review and were screened for the following secondary inclusion and exclusion criteria. Secondary inclusion criteria included (1) SMS, IVR, or CATI was used for data collection and (2) MPS was intended to be population-representative. Secondary exclusion criteria included (1) study was not conducted in a LMIC as defined by the World Bank [12], (2) respondents were interviewed in-person by an enumerator using a mobile phone, and (3) surveys were sent to respondent’s landline telephone number. Records were included independent of the survey’s content (ie, health, agriculture, economics, and so on). Articles that were written in languages other than English were not reviewed.

Two research assistants extracted data from all included articles. Disagreements in data extraction were resolved by consensus between the two research assistants. Data were entered into an Excel (Microsoft) worksheet and grouped by survey modality to populate tables. For panel surveys where the same respondent answered a series of surveys over time, the response, completion, and refusal rates from the first round of surveys were abstracted and presented in the identified manuscripts.

## Results

### Overview

**Figure 1:**
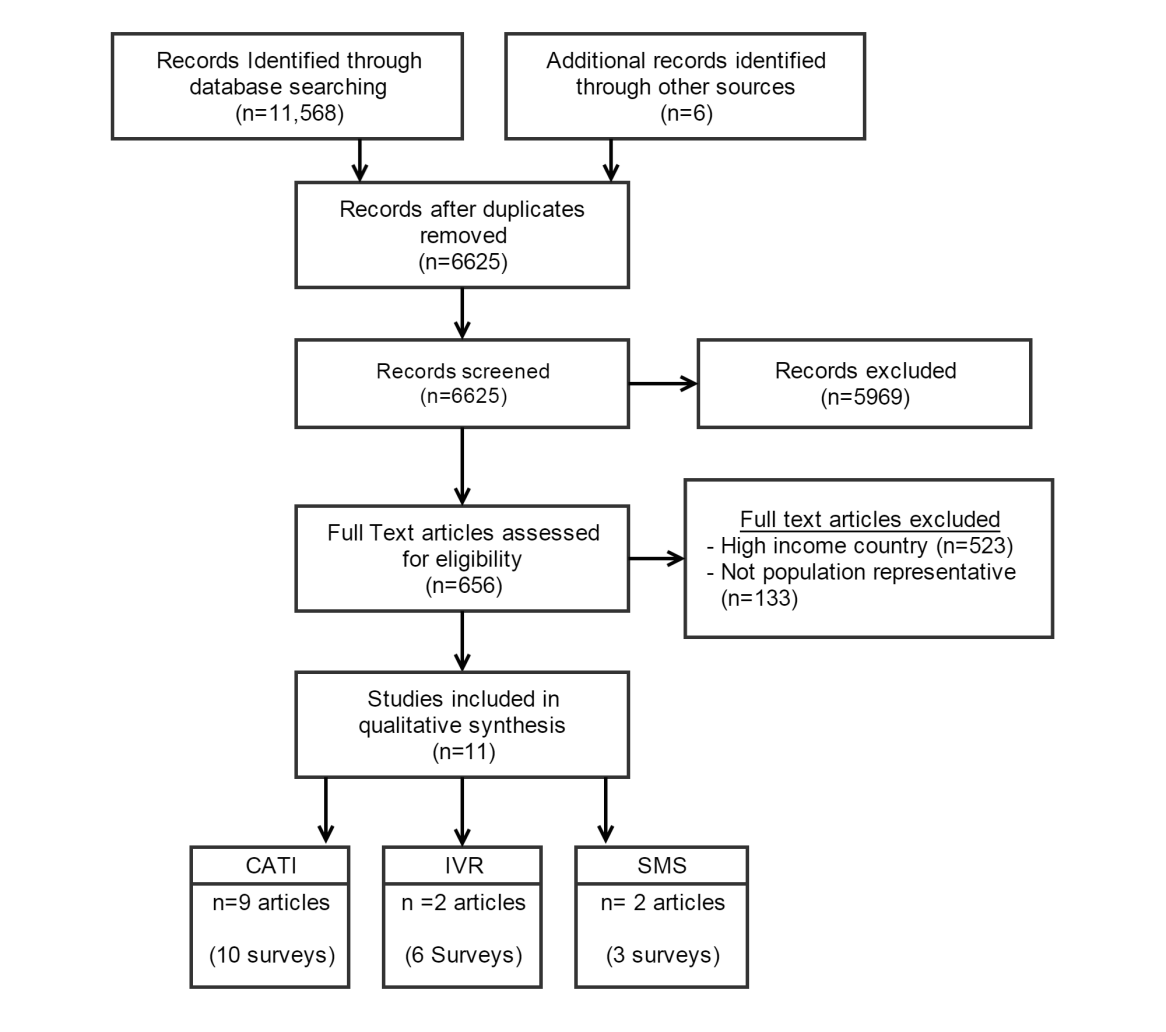
Flow diagram of the study. CATI: computer-assisted telephone interview; IVR: interactive voice response; SMS: short message service. One article included surveys for CATI (n=2), IVR (n=2), and SMS (n=2).

The literature search identified 11,568 records. After removing duplicates, 6625 records underwent a primary screening of titles and abstracts (Figure 1). Full-text articles (n=656) were then screened using secondary inclusion and exclusion criteria. Overall, we identified 11 articles that employed 19 MPS (CATI, IVR, or SMS) surveys to collect population estimates across a range of topics. Nine articles presented results for 10 CATI surveys (10/19, 53%) [13-21]. Two articles discussed the findings of 6 IVR surveys (6/19, 32%) [13,22]. Three SMS surveys were identified from 2 articles (3/19, 16%) [13,23].

### CATI Surveys

The majority of MPS implemented in LMICs were conducted by human interviewers, typically stationed at call centers equipped with CATI software (Table 1). The locations and questionnaire topics were diverse; CATI surveys were conducted in Bangladesh, Brazil, Honduras, Lebanon, Liberia, Mali, Peru, South Sudan, and Tanzania and covered topics on health and socioeconomics.

Of the 10 identified CATI surveys, 60% (6/10) were implemented through the World Bank or as part of the Listening to Africa (L2A) and Listening to Latin America and the Caribbean (L2LAC) Initiatives [13,14,16-18]. In these initiatives, a population-representative sample of households was drawn and a baseline household visit was made; survey staff interviewed the selected household member in-person and provided training on how to answer future mobile phone panel surveys (Panel MPS). Panel MPS were typically sent monthly to collect information on general welfare questions, such as household assets, food security, and employment [24]. The literature review identified one Panel MPS in Tanzania that was not affiliated with the World Bank and L2A or L2LAC [15]. The number of survey rounds or waves ranged from 2 to 33 with a typical interval of 3-6 weeks between each wave. In 71% (5/7) of the Panel MPS, mobile phones were provided to all participants [14-16] or only provided to those who did not already own one [13].

Three studies employed a cross-sectional CATI survey, rather than a panel MPS [19-21]. Mobile phones were not provided to any of these participants. The Lebanese survey sampled participants who had provided a phone number during the nationwide Nutrition and Noncommunicable Disease Risk Factor Survey [20]. The median time between the household and CATI survey was 1.8 months. In Bangladesh and Brazil, participants for noncommunicable diseases (NCD) risk factor surveys were sampled from a list of subscribers provided by a mobile network operator (MNO) [19] or through random digit dialing (RDD), respectively [21]. Since 2006, Brazil’s Ministry of Health has conducted annual telephone surveys for risk and protective factors of NCD. Articles that presented VIGITEL surveys where the sampling frame contained only landline telephone numbers were excluded as the purpose of this review was to document MPS [25-29].

Overall, the response rates and completion rates for CATI surveys were highly variable, ranging from 30% to 98% and from 35% to 100%, respectively, although completion rates were only presented in 30% (3/10) of surveys. It is likely that for studies that did not report, the completion rate may near 100% as one study commented that it is the interviewer’s job to make sure all questions are answered [13]. In the three studies that reported refusal rate, estimates ranged from 2% to 8% [17,20,21]. For Panel MPS, typically, panel attrition was highest at the first CATI following the household baseline survey, with attrition and nonresponse rates plateauing over the duration of the panel.

Varying airtime incentive amounts, tied to survey completion, were randomized in 40% (4/10) of CATI surveys [13,14,18], all of which were Panel MPS, to evaluate their effect on survey response and completion rates. In two surveys that did not contain a control arm (ie, no incentive), there was either no discernible effect between the low and high incentive amount on response rates [18], or the higher incentive arm had lower response rate as compared with the lower incentive arm [14]. In Peru and Honduras, panelists were randomized to one of the following three arms: (1) no incentive, (2) US $1 airtime, and (3) US $5 airtime [13]. In Honduras, both incentive arms significantly improved survey response throughout the panel, as compared with the control arm. In Peru, results were not disaggregated by survey modality (CATI, IVR, and SMS). The authors reported no appreciable difference in the first survey’s response rate by the study arm; with similar gains in the two incentive arms at minimizing panel attrition over the duration of the study. Of note, the study’s authors indicate that the incentive arm contained the majority of people who were provided a study-sponsored mobile phone. An additional three panel surveys provided a fixed US $1-2 airtime incentive to all panelists [15-17]. Incentives were not used in the three cross-sectional surveys [19-21].

**Table 1:** Computer-assisted telephone interviews (CATI) or human operator-administered surveys (n=10 surveys, 9 articles).

Author	Country (sample size)	Survey type	Sampling frame	Phone given	Response %^a^ (completion %)	Average time to complete (# questions)
Ballivian et al [13]	Peru (n=384)	Panel (n=6 waves)	Household collected	If not owned	51% 100%	(10)
	Honduras (n=600)	Panel (n=2 waves)	Household collected	If not owned	88%	(10)
Demombynes et al [14]	South Sudan (n=1007)	Panel (n=4 waves)	Household collected	Yes	69%	15-20 min (16-26)
Dillon [15]	Tanzania (n=195)	Panel (n=14 waves)	Household collected	Yes	98% overall	≈27 min
Etang-Ndip et al [16]	Mali (n=501)	Panel (n=6 waves)	Household collected	Yes	99%	20-25 min
Himelein [17]	Liberia (n=2137)	Panel (n=2 waves)	Household collected	No	30%	≈15 min
Hoogeven et al [18]	Tanzania (n=458)	Panel (n=33 waves)	Household collected	No	75% overall	19 min
Islam et al [19]	Bangladesh (n=3378)	Cross-sectional	Mobile network operator	No	61%	
Mahfoud et al [20]	Lebanon (n=771)	Cross-sectional	Household collected	No	(82%)	8 min
Moura et al [21]	Brazil (n=1207)	Cross-sectional	Random digit dialing	No	(≈35%)	5 min

^a^For panel surveys, the response, completion, and refusal rates listed are for the first round of MPS unless otherwise indicated.

**Table 2:** IVR-administered surveys (n=6 surveys, 2 articles).

Author	Country (sample size)	Survey type	Sampling frame	Phone given	Response %^a^ (completion %)	Average time to complete (# (questions)
Ballivian et al [13]	Peru (n=383)	Panel (n=6 waves)	Household collected	If not owned	20% 75%	(10 Q)
	Honduras (n=600)	Panel (n=2 waves)	Household collected	If not owned	40%	(10 Q)
Leo et al [22]	Afghanistan (n=2123)	Cross-sectional	Random digit dialing	No	31% (30%)	4-5 min (10 Q)
	Ethiopia (n=2258)	Cross-sectional	Random digit dialing	No	19% (23%)	4-5 min (10 Q)
	Mozambique (n=2229)	Cross-sectional	Random digit dialing	No	9% (38%)	4-5 min (10 Q)
	Zimbabwe (n=2192)	Cross-sectional	Random digit dialing	No	8% (51%)	4-5 min (10 Q)

^a^For panel surveys, the response, completion, and refusal rates listed are for the first round of MPS unless otherwise indicated.

### IVR Surveys

MPS that employed IVR were less frequently covered in existing literature (Table 2). Our literature review identified two articles that described the findings of 6 IVR surveys to collect population-level estimates [13,22]. One article employed a standardized methodology across 4 countries—Afghanistan, Ethiopia, Mozambique, and Zimbabwe—to collect demographic and standard of living information [22]. Participants were selected through RDD with a demographic quota system and were not provided a mobile phone. The remaining 2 IVR surveys were conducted in Honduras and Peru as part of the L2LAC initiative, where participants had previously completed a baseline household survey and were provided a mobile phone as needed [13]. All 6 surveys utilized a 10-question survey; 4 surveys reported that respondents, on average, interacted with the survey for 2-3 min and for those who completed the 10-question survey, it took between 4 and 5 min [22]. Response rates were typically higher for IVR surveys that were sent to mobile phone numbers collected from a previous household survey (20% and 40%) than from those using an RDD approach (8%, 9%, 19%, and 31%).A wide range (23-75%) of survey completion rates was observed.

The effect of airtime incentives to improve survey response and completion rates [22] and panel attrition rates [13] was evaluated across the 6 surveys and produced mixed results. One article randomized RDD participants to a control arm, 4-min airtime incentive transfer, and a raffle for a 2-h airtime incentive; where participants in the two airtime arms were eligible for the incentive if the survey was completed [22]. In Zimbabwe, the transfer and raffle incentives significantly improved the proportion of participants who completed the survey; while in Mozambique, only the raffle incentive was found to be significant. A similar evaluation was conducted in Afghanistan and Ethiopia, but the authors commented that there were problems with the randomization and allocation of study arm. In Honduras, those who were randomized to either US $1 or US $5 of airtime incentive showed higher response rates than those who did not receive an incentive [13]. As described previously, response rates were not disaggregated by survey modality in Peru.

### SMS Surveys

Although data collection via SMS surveys is relatively common in LMICs, very few studies aimed to collect data on a representative sample of a population (Table 3). One study sampled 982,708 phone numbers from a network of 18 million prepaid mobile phone subscribers in Mexico to participate in a surveillance program regarding influenza-like illness [23]. Mobile phone subscribers were sent a text message from the Ministry of Health, inviting them to participate in a 6-question survey. The surveillance program resulted in a 5.8% response rate. The mean age of respondents was 25 years and nearly 90% of surveys were completed within 24 h of the initial contact. No incentives were provided.

As part of the previously described L2LAC, SMS surveys were also deployed in Peru and Honduras to collect population representative estimates [13]. The response rates for the first round of SMS surveys were 30% and 45% in Peru and Honduras, respectively. Approximately 80% of participants completed the ten question survey in Peru. In Honduras, providing either US $1 or US $5 of airtime significantly improved response rate, as compared with those who did not receive any airtime incentive.

### Comparison of Survey Metrics Across Different MPS Modalities

Only two surveys compared key survey metrics such as response and completion rates across MPS. The response rate for the first round of Panel MPS was highest for CATI (Honduras, 88%; Peru, 51%), followed by SMS (45%; 30%) and IVR (40%; 20%) [13]. In Peru, CATI showed a 100% completion rate; with completion rates of 80% and 75% in SMS and IVR surveys, respectively. In the same set of surveys, the reliability of the respondent’s answer was assessed through a test-retest procedure. Cronbach alpha coefficient for CATI, IVR, and SMS were .69, .86, and .74, respectively, indicating that IVR resulted in the most reliable measurements. Of note, such survey metrics have not been compared across survey modalities using sampling frames other than household collected phone numbers (eg, RDD or MNO provided).

### Excluded Studies

Several large SMS surveys were identified but were excluded because they did not seek to attain representativeness. Two SMS surveys recruited participants through social media platforms. [30,31] Demographic information regarding the users of these platforms was not included thus making it difficult to assess the representativeness of respondents. Two studies attempted to achieve a subnational sample through opt-in recruitment, potentially introducing selection bias [32,33]. Numerous studies used SMS and IVR surveys as a data collection tool within a research study [34-54] or as a surveillance instrument for health care workers [55-59] and were excluded from the review.

## Discussion

### Principal Findings

Our literature review identified very few reports of MPS being used to collect population-level estimates. CATI surveys (n=10), most frequently relying on a household baseline survey to collect mobile phone numbers and implemented by the World Bank, were the most common type of MPS reported. When there was a household collection of mobile phone numbers, frequently, the implementing team conducted panel surveys (repeated MPS to the same respondent over time).

**Table 3:** SMS-administered surveys (n=3 surveys, 2 articles).

Author	Country (sample size)	Survey type	Sampling frame	Phone provided	Response %^a^ (completion %)	Average time to complete (# questions)
Ballivian et al [13]	Peru (n=677)	Panel (n=6 waves)	Household collected	If not owned	30% 80%	(10 Q)
	Honduras (n=600)	Panel (n=7 waves)	Household collected	If not owned	45%	(10 Q)
Lajous et al [23]	Mexico (n=982,708)	Cross-sectional	Mobile network operator	No	6%	(6 Q)

^a^For panel surveys, the response, completion, and refusal rates listed are for the first round of MPS unless otherwise indicated.

The selection of the MPS modality has important downstream impacts on costs, survey metrics, and data quality, with each modality having its strengths and weaknesses [13,14] (Table 4). Evidence from one study that compared costs across the three modalities found that SMS and IVR surveys are less expensive than CATI surveys [13]. The primary cost of IVR and SMS surveys are airtime needed to deliver the survey, with additional costs for initial programming and monitoring survey delivery. CATI surveys, in addition to the cost of airtime and programming, also require personnel—human interviewers and supervisors—to conduct the survey, making their delivery more costly than IVR or SMS surveys [13]. The higher costs of CATI surveys are partially offset by the advantage of having a human to conduct the survey. This offers an opportunity for personalized responses to clarify any confusion a respondent may have, potentially resulting in higher quality data and lower levels of survey attrition. This benefit is supported from surveys in Peru and Honduras where response and completion rates were highest for CATI, as compared with IVR and SMS surveys. Additional studies that use a standardized approach to examine the effect of survey modality on survey response, completion, and refusal rates are needed.

**Table 4:** Strengths and weaknesses of mobile phone surveys (MPS) by modality (adapted from Demombynes (2013) and Ballivian (2013)).

Strengths		Weaknesses
**Computer-assisted telephone interview (CATI^)^**		
	Respondent’s familiarity with a phone call interaction	Resource intensive (operators, supervisors, training)
	Operators can clarify questions	Inter-rater reliability concerns
	Ability to build rapport with respondents	Potential for interviewer bias
	Does not require respondents to be literate	Respondents may be less truthful for sensitive questions
		Requires sustained network signal
**Interactive voice Response (IVR)**		
	Mimics a phone call	Requires sustained network signal
	Does not require respondents to be literate	Respondents may not be familiar with “robot” calls
	Automated surveys allows for quick data collection	Potential for respondent to be distracted while answering the survey
	Minimizes interviewer bias	Poor audio quality of some phones
	Less expensive than CATI due to its automation	
**Short Message Service (SMS)**		
	Respondents answer at their convenience	May not reach illiterate respondents
	Automated surveys allows for quick data collection	Requires network signal, possibility of lost messages
	Minimizes interviewer bias	Question length limited by character count
	Less expensive than CATI due to its automation	Inbox can become full

The majority of identified studies relied on household-collected mobile phone numbers as the sampling frame [13-18,20]. Like the MPS modality, the choice of sampling frame has implications on cost, key survey metrics, and potential representatives of a MPS. In an RDD approach there will be a significant proportion of randomly generated telephone numbers that do not exist or are not registered [60]; this represents an added cost, particularly for CATI surveys and their reliance on human operators, as more telephone calls need to be made in order to achieve the survey’s sample size. Similarly, and dependent on the equation used for calculation [61], the response and completion rates may appear to be artificially lower in an RDD sample as compared with sampling frames, such as household collected or MNO-provided ones, which ensure that the mobile phone numbers collected are active.

The use of incentives to improve response and completion (ie, cooperation) rates in telephone and postal surveys in high-income countries is well-documented [62]. Similarly, incentivizing participants through the provision of free airtime has the potential to increase the response and completion rates and the demographic representativeness of MPS, yet the findings from the few randomized trials provides inconclusive evidence on whether these interventions are effective [13,14,18,22]. Additional research studies on the use of airtime incentives and other mechanisms to improve survey performance and outcomes are needed.

Access to a mobile phone and mobile network coverage are implicit factors in a MPS’s ability to generate population-representative estimates. Moreover, the “digital divide” phenomenon, where mobile phone ownership is associated with socioeconomic status, may also pose challenges with obtaining representative estimates—although evidence suggests this divide is shrinking [63]. To increase the likelihood of a survey’s representativeness, household sampling methodologies can be applied to obtain a sample of household-collected mobile phone numbers. However, this requires an initial investment of human and financial resources to collect the phone numbers and is more appropriate for cohort studies or panel surveys where the initial investment will be recouped with each subsequent survey. An RDD sampling frame is more suitable for cross-sectional surveys and is the standard sampling approach for telephone surveys [60]. Our review identified very few MPS that employed RDD, but the evidence suggests that it is feasible to obtain a representative sample and that it is dependent on the saturation levels of mobile phone ownership and, to a lesser extent, linguistic fractionalization [22]. Still, options exist for obtaining population-representative results using RDD [64].

### Limitations

The literature review and its inclusion and exclusion criteria identified very few articles that employed SMS (n=2 articles) or IVR surveys (n=2) to collect population representative estimates. There are three potential reasons for the infrequent use of IVR and SMS surveys. First, the search terms used in our literature review did not identify all relevant articles. Second, the pilot testing results from the L2A and L2LAC initiatives may have artificially driven the overrepresentativeness of CATI surveys identified in our literature review. Before the implementation of the initiatives, a series of pilot tests identified that CATI surveys yielded higher completion rates than IVR and SMS surveys; leading the World Bank to adopt the CATI modality as its preferred survey. Thirdly, the MPS field is in its infancy phase and there may truly be very few reports of attempts at population-representative surveys using IVR and SMS. An additional limitation is the presentation of the response and completion rates. The majority of the manuscripts did not present the equations used to calculate these rates, as recommended by the American Association for Public Opinion Research [61].

### Conclusions

In conclusion, the state of MPS to collect population level estimates of health and other indicators remains nascent. Additional research that directly compares the costs, key survey metrics such as contact, response, completion, and refusal rates, and demographic representativeness across the different survey modalities is needed [10]. Still, if MPS are found to produce valid and reliable data, their use has the potential to compliment traditional household surveys and benefit existing surveillance efforts by leveraging their lower costs to allow for a more frequent monitoring of the population’s health.

## Appendix

Multimedia Appendix 1 Examples of search terms used.

## Abbreviations

CATI: computer-assisted telephone interviews
IVR: interactive voice response
L2A: Listening to Africa
L2LAC: Listening to Latin America and the Caribbean
LMIC: low- and middle-income countries
MNO: mobile network operator
MPS: mobile phone surveys
NCD: noncommunicable diseases
PRISMA: Preferred Reporting Items for Systematic Reviews and Meta-Analyses
RDD: random digit dialing
SMS: short message service
