# Supplementary material for: Mobile Phone Surveys for Collecting Population-Level Estimates in Low- and Middle-Income Countries: A Literature Review
Source: J Med Internet Res. 2017 May 5;19(5):e139. doi: 10.2196/jmir.7428 (PMC5438460; doi:10.2196/jmir.7428)
Supplement: Multimedia Appendix 1 [file jmir_v19i5e139_app1.pdf]

Appendix 1. Example of search terms used

(("cell phone" OR “mobile phone”) AND (survey OR questionnaire* OR “data collection”)) OR

(("IVR" OR “interactive voice response”) AND (survey OR questionnaire* OR “data collection”)) OR

(("SMS" OR “short message service” OR (text AND message*)) AND (survey OR questionnaire* OR “data collection”))
